# Leptin and insulin up-regulate miR-4443 to suppress NCOA1 and TRAF4, and decrease the invasiveness of human colon cancer cells

**DOI:** 10.1186/s12885-016-2938-1

**Published:** 2016-11-14

**Authors:** Ari Meerson, Hila Yehuda

**Affiliations:** 1 Molecular Biology of Chronic Diseases, MIGAL Galilee Research Institute, PO Box 831, Kiryat Shmona, 11016 Israel; 2 Department of Biomedical Sciences, University of Copenhagen, Blegdamsvej 3, Copenhagen, DK-2200 Denmark

**Keywords:** Obesity and cancer, Leptin resistance, Non-coding RNA, Cancer cell lines, Invasion, Proliferation, Cell culture

## Abstract

**Background:**

Obesity is a risk factor for colorectal cancer (CRC). Normal and tumor cells respond to metabolic hormones, such as leptin and insulin. Thus, obesity-associated resistance to these hormones likely leads to changes in gene expression and behavior of tumor cells. However, the mechanisms affected by leptin and insulin signaling in CRC cells remain mostly unknown.

**Methods:**

We hypothesized that microRNAs (miRNAs) are involved in the regulation of tumorigenesis-related gene expression in CRC cells by leptin and insulin. To test this hypothesis, miRNA levels in the CRC-derived cell lines HCT-116, HT-29 and DLD-1 were profiled, following leptin and insulin treatment. Candidate miRNAs were validated by RT-qPCR. Predicted miRNA targets with known roles in cancer, were validated by immunoblots and reporter assays in HCT-116 cells. Transfection of HCT-116 cells with candidate miRNA mimic was used to test in vitro effects on proliferation and invasion.

**Results:**

Of ~800 miRNAs profiled, miR-4443 was consistently up-regulated by leptin and insulin in HCT-116 and HT-29, but not in DLD-1, which lacked normal leptin receptor expression. Dose response experiments showed that leptin at 100 ng/ml consistently up-regulated miR-4443 in HCT-116 cells, concomitantly with a significant decrease in cell invasion ability. Transfection with miR-4443 mimic decreased invasion and proliferation of HCT-116 cells. Moreover, leptin and miR-4443 transfection significantly down-regulated endogenous NCOA1 and TRAF4, both predicted targets of miR-4443 with known roles in cancer metastasis. miR-4443 was found to directly regulate TRAF4 and NCOA1, as validated by a reporter assay. The up-regulation of miR-4443 by leptin or insulin was attenuated by the inhibition of MEK1/2.

**Conclusions:**

Our findings suggest that miR-4443 acts in a tumor-suppressive manner by down-regulating TRAF4 and NCOA1 downstream of MEK-C/EBP-mediated leptin and insulin signaling, and that insulin and/or leptin resistance (e.g. in obesity) may suppress this pathway and increase the risk of metastatic CRC.

**Electronic supplementary material:**

The online version of this article (doi:10.1186/s12885-016-2938-1) contains supplementary material, which is available to authorized users.

## Background

Obesity and cancer are leading health problems in developed countries. Cancer is the consequence of inherited and somatic mutations that is also influenced by the physiological micro- and macro-environment (e.g. obesity). A positive association has been found between obesity and the risk for the development of various cancers, among them colorectal cancer (CRC) [1, 2]. Several mechanisms have been proposed to explain this association: chronic inflammation, excess production of leptin [3] (concomitantly with an onset of systemic resistance to leptin signaling [4]) and decreased adiponectin secretion in obese subjects, which may deregulate cellular growth and angiogenesis, and therefore promote cancer development and progression [5–8].

Although the contributions of many genes are beginning to emerge as important links between obesity and cancer, much research is still needed to better understand the complexity of gene regulation. The expression of genes is regulated, in part, by microRNAs (miRNAs), endogenous small non-coding RNAs (ncRNAs), which play regulatory roles in normal cell function and in diseases, especially cancers, by predominantly binding to cis-elements in the 3′ untranslated region of specific mRNAs and regulating their translation or stability [9–13]. Recent studies have begun to elucidate the role of miRNAs in various biological processes, including adipocyte differentiation, metabolic integration, insulin resistance and appetite regulation [14–17]. The deregulation of many miRNAs in metabolic tissues of obese animals and humans has also been described [15, 16, 18].

The involvement of miRNAs in cancer, and specifically CRC, is well known (reviews, [19–22]). Interestingly, a number of miRNAs that are associated with obesity have also been implicated in carcinogenesis, and deregulated expression of miRNAs may represent a molecular link between obesity and cancer [23]. Here, we show that a specific miRNA, miR-4443, responds to leptin and insulin treatment in CRC-derived cells and that its impaired regulation may contribute to deregulation of downstream signaling, increased cancer metastasis and worse prognosis in a state of leptin resistance.

## Methods

We hypothesized that microRNAs (miRNAs) are involved in the regulation of tumorigenesis-related gene expression in CRC cells by leptin and insulin. To test this hypothesis, miRNA levels in the CRC-derived cell lines HCT-116, HT-29 and DLD-1 were profiled, following leptin and insulin treatment. Candidate miRNAs were validated by RT-qPCR. Predicted miRNA targets with known roles in cancer, were validated by immunoblots and reporter assays in HCT-116 cells. Transfection of HCT-116 cells with candidate miRNA mimic was used to test in vitro effects on proliferation and invasion.

### Cells and cell culture

CRC-derived cell lines DLD-1, HT29 and HCT116 were provided by Professors Ronit Pinkas-Kramarski, Rimona Margalit and Yoel Kloog from Tel Aviv University in September 2013, and kept frozen in liquid nitrogen until use. Cell line authentication was performed at the Biomedical Core Facility of the Rappaport faculty of Medicine, Technion. Cells were cultured based on ATCC recommendations and treated with the 20, 100, or 200 ng/ml (as indicated) of leptin (Sigma L4146) or insulin (Sigma I2643). Culture media and fetal bovine serum were obtained from Biological Industries (Israel). PD-98059 (Adipogene), dissolved in 100 % DMSO, was added to culture medium (final concentration 10 μM PD-98059 and 0.04 % DMSO), 45 min before the addition of leptin or insulin.

### Proliferation and invasion assays

Cell proliferation was determined 48 h after transfection and 3 days after leptin or insulin treatment, using the CyQUANT Direct Cell Proliferation Assay (Life Technologies) in a 96-well plate format in a Tecan Infinite M200 Pro spectrophotometer. Invasion of HCT-116 cells was measured after 48 h using Matrigel (BD) in inserts with 8 μm pores (Greiner), placed in 24-well cell culture plates (Biological Industries). Cells were collected from both upper and lower chambers by trypsinization and evaluated using CyQUANT. Invasion was calculated by dividing the measurement of the cells in the lower chamber (that had passed through Matrigel) by the total measurement of cells from both upper and lower chambers, and this value was normalized to migration values (obtained without Matrigel) calculated similarly. The resulting values were then normalized to the appropriate control values.

### miRNA target prediction

A list of predicted miR-4443 targets was obtained using the TargetScan algorithm [9, 24] (Release 6.2), accessible online [25].

### Immunoblots

BioRad equipment and reagents were used. Primary antibodies were from Abcam: human NCOA1/KAT13/SRC1 (AB2859) [26], human TRAF4 (AB190986, at 0.4 μg/ml in PBS), human β-actin (AB6276) [27], and human leptin receptor (AB104403 at 0.3 μg/ml in PBS). Secondary HRP-conjugated antibodies were also from AbCam. Bands were visualized on film using SuperSignal™ West Pico Chemiluminescent Substrate (Thermo Scientific). ImageJ software (NIH) [28] was used for image analysis.

### RNA isolation

Isolation of total RNA (including miRNAs) was carried out using the Qiagen miRNeasy Kit according to manufacturer’s instructions.

### miRNA profiling

Expression profiling of miRNAs was performed on an nCounter probe array platform (Nanostring). Hierarchical clustering of results was performed using MeV, part of the TM4 software suite [29, 30].

### RT-qPCR

Reverse transcription, primer design and quantitative PCR were performed using SYBR Green chemistry and DNA primers. Primer extension was used for miRNA quantification as previously described [31, 32]. This RT-qPCR method is highly accurate and reproducible [31, 32], and was chosen over hydrolysis probe chemistry due to lower cost and higher convenience. Primer sequences are provided in Additional file 1: Table S1. All primers were tested for efficiency (by serial dilutions) and specificity (by melting peak analysis). RT was performed on a ABI-9600 with reagents from New England Biolabs. qPCR was performed in technical quadruplicates on an Applied Biosystems ABI-7900HT Sequence Detection System equipped with a 384-well block. The biological sample sizes were as indicated in the figure legends. Data were analyzed using SDS 2.3 software (Applied Biosystems) and Microsoft Excel. Relative quantification and the ΔCq method were used. For quantification of mRNAs, β-actin was used as a normalizer. For quantification of miRNAs, normalization was performed relative to the average values from a panel of at least 5 miRNAs that previously showed no significant change following treatment.

### Transfection

HCT-116 cells were transfected with Dharmacon miRIDIAN mimic of miR-4443 and the negative control oligo, as well as reporter vectors, using DharmaFECT 4 transfection reagent, in 6-well, 24-well or 96-well plates depending on intended assay, according to the manufacturer’s instructions. For 3′ UTR reporter assays, Genecopoeia SecretePair dual reporter constructs for TRAF4, NCOA1 and a no-UTR control vector, co-transfected with miRNA mimics, were used.

### Reporter assay

Luciferase activity was measured in a 96-well plate format in a Tecan Infinite M200 Pro spectrophotometer.

### *In silico* promoter analysis

Promoter/enhancer region cis-element prediction was carried out employing the Cister algorithm [33] available online [34]. A 10 kb segment of the human genomic sequence upstream of the miR-4443 locus (3:48186564–48196564) was used, having ascertained that the segment contained no other known genes in the “plus” orientation.

### Statistics

For statistical tests, Student’s *t*-test was used. For correlations, linear regression was used.

## Results

### Leptin and insulin up-regulate miR-4443 in CRC-derived cell lines; the effect of leptin on miR-4443 is LEPR-dependent

**Fig. 1:**
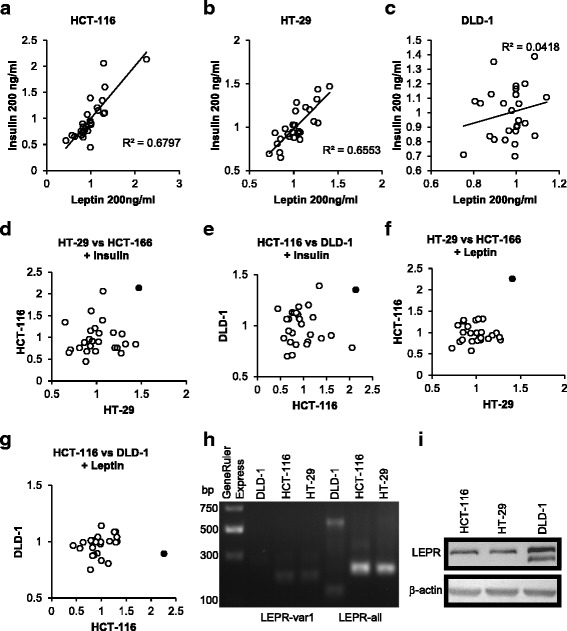
Leptin has effects similar to those of insulin on miRNA expression profiles in CRC-derived cell lines, and these effects are LEPR-dependent. **a** Scatter plot comparing the effects of leptin and insulin (both at 200 ng/ml), on the expression (relative to non-treated controls) of individual miRNAs as quantified by Nanostring profiling, in HCT-116 cells. **b** Scatter plot as in (**a**), in HT-29 cells. **c** Scatter plot as in (**a**), in DLD-1 cells. **d** Scatter plot comparing the effects of 200 ng/ml insulin on the expression (relative to non-treated controls) of individual miRNAs as quantified by Nanostring profiling, in HT-29 and HCT-116 cells. Circle representing miR-4443 marked in black. **e** Scatter plot as in (**d**), in HCT-116 and DLD-1 cells. Circle representing miR-4443 marked in black. **f** Scatter plot comparing the effects of 200 ng/ml leptin on the expression (relative to non-treated controls) of individual miRNAs as quantified by Nanostring profiling, in HT-29 and HCT-116 cells. Circle representing miR-4443 marked in black. **g** Scatter plot as in (**f**), in HT-116 and DLD-1 cells. Circle representing miR-4443 marked in black. **h** Electrophoresis of PCR products obtained with LEPR primers targeting a conserved region (common to all known variants), as well as specific to the longer and active variant of LEPR, and cDNA from DLD-1, HCT-116, and HT-29 cells. **i** Immunoblot for human LEPR in HCT-116, HT-29 and DLD-1 cells. β-actin was used as loading control

To check the effects of leptin and insulin exposure on the expression levels of a wide cross-section of miRNAs, we profiled miRNA levels in the CRC-derived cell lines HCT-116, HT-29 and DLD-1 that had been treated with leptin or insulin (at 200 ng/ml) for 24 h, using the Nanostring nCounter probe array platform (clustered heat map for leptin-induced changes, Additional file 2: Figure S1; all expression data, Additional file 3: Table S2). A positive correlation between the effects of insulin and leptin on miRNA expression profiles was observed in HCT-116 and HT-29 cells, but not in DLD-1 (Fig. 1a–c). Additionally, the effects of insulin on miRNA expression profiles correlated in all three cell lines (Fig. 1d, e) but the effects of leptin only correlated between HCT-116 and HT-29, but not between them and DLD-1 (Fig. 1f, g). Of ~800 miRNAs profiled, miR-4443 stood out in its robust and similar response to leptin and insulin. Thus, miR-4443 was up-regulated by insulin in all three lines, and by leptin in HCT-116 and HT-29, but not in DLD-1 (miR-4443 marked in black, in Fig. 1d–g). These results suggest that HCT-116 and HT-29 express the functional leptin receptor (LEPR), which is necessary for leptin’s downstream effects on the miRNA profile, while DLD-1 may lack the receptor or express an inactive form of LEPR. RT-qPCR using primers targeting the domain common to all variants of LEPR yielded a specific product at the expected length for HCT-116 and HT-29 – derived DNA, but that of DLD-1 was shorter (Fig. 1h). Additionally, no PCR product was observed for DLD-1, when primers specifically targeting LEPR variant 1 (the long and active form) were employed (Fig. 1h). This lack of a full-length LEPR transcript was confirmed in 2 separate stocks of DLD-1 cells, from different sources (data not shown). Immunoblots for LEPR produced two bands in DLD-1, the upper one rather strong, and both differing in size from the single band in the other cell lines (Fig. 1i), suggesting the accumulation of altered or inactive LEPR variants in DLD-1. We chose HCT-116 for further study as this cell line was morphologically and phenotypically stable in culture and was confirmed to express LEPR.

### Leptin up-regulates miR-4443 and decreases invasion in HCT-116 cells

**Fig. 2:**
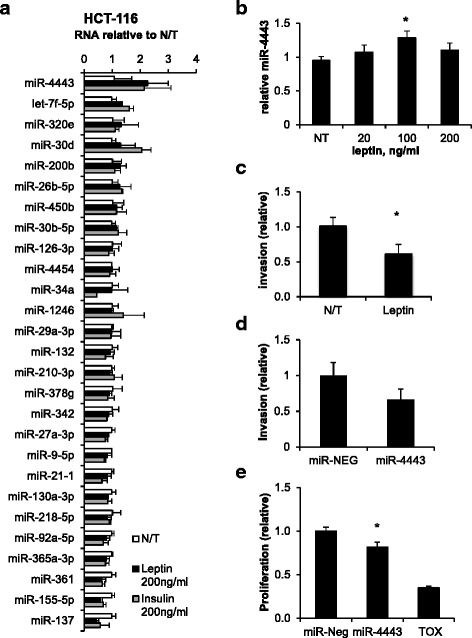
Leptin exposure up-regulates miR-4443 and decreases invasion, while overexpression of miR-4443 decreases the proliferation of cultured HCT-116 cells. **a**. RT-qPCR validation of the effects of leptin and insulin, both at 200 ng/ml, on the expression of candidate miRNAs identified by Nanostring profiling, in HCT-116 cells. Bars, SD from triplicates. **b** Dose response of miR-4443 levels under leptin exposure, in HCT-116 cells. *:*p* < 0.05; *t*-test; bars: SD from triplicates. **c** CyQuant quantification of Matrigel invasion assay over 48 h, in HCT-116 cells with or without 100 ng/ml leptin. *:*p* < 0.05; *t*-test; bars: SE; *n* = 6. **d** CyQuant quantification of Matrigel invasion assay over 48 h, in HCT-116 cells transfected with miR-4443 mimic or negative control (miR-Neg). bars: SE; *n* = 5. **e** CyQuant quantification of cell proliferation over 48 h, in HCT-116 cells transfected with miR-4443 mimic or negative control (miR-Neg). Cell death-causing TOX oligo was used as transfection control. *:*p* < 0.05; *t*-test; bars: SE; *n* = 9

The Nanostring profiling results of miRNA levels obtained from HCT-116 cells treated with leptin or insulin at 200 ng/ml were validated by RT-qPCR (Fig. 2a). miR-4443, which showed robust up-regulation by leptin in both the Nanostring profiling and the qRT-PCR validation, was chosen as a candidate for further study. In a dose-response experiment, leptin treatment at 100 ng/ml was found to up-regulate miR-4443 reproducibly as well as significantly (*p* < 0.05) (Fig. 2b). This dose of leptin also caused a significant (*p* < 0.05) decrease in cell invasion through a MatriGel-coated membrane (Fig. 2c). We therefore used this dosage of leptin for further experiments. We also established that 20 ng/nl insulin was sufficient to cause the up-regulation of miR-4443 (data not shown).

### Overexpression of miR-4443 decreases the invasion and proliferation of HCT-116 cells

Transfection of HCT-116 with a miR-4443 mimic tended to decrease cell invasion as compared to transfection with a control oligo (Fig. 2d); this trend was reproducible but fell short of statistical significance due to large variation between individual experiments. Overexpression of miR-4443 also significantly decreased the proliferation of HCT-116 cells (Fig. 2e), implying that miR-4443 may act as a tumor suppressor via its downstream targets.

### Leptin and insulin exposure and miR-4443 overexpression down-regulate NCOA1 and TRAF4 in HCT-116 cells

**Fig. 3:**
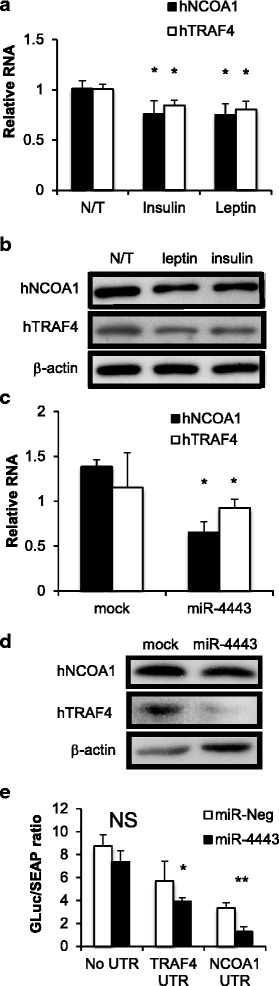
Leptin and insulin exposure and overexpression of miR-4443 down-regulate the miR-4443 targets, NCOA1 and TRAF4, in HCT-116 cells. **a** RT-qPCR for human NCOA1 and TRAF4 in HCT-116 cells cultured for 48 h with or without 100 ng/ml leptin or 20 ng/ml insulin. *:*p* < 0.05; *t*-test; bars: SD from triplicates. **b** Immunoblot for human NCOA1 and TRAF4 in HCT-116 cells as in (a). β-actin was used as loading control. **c** RT-qPCR for human NCOA1 and TRAF4 in HCT-116 cells transfected with miR-4443 mimicking oligo, or mock-transfected cells. *:*p* < 0.05; *t*-test; bars: SD, *n* = 5. **d** Immunoblot for human NCOA1 and TRAF4 in HCT-116 cells transfected with miR-4443 mimicking oligo, or mock-transfected cells. β-actin was used as loading control. **e** Secrete-Pair Luciferase/SEAP ratios in media of HCT-116 cells 48 h post co-transfection with constructs containing the 3′ UTR of TRAF4, NCOA1, or no UTR, with miR-4443 - mimicking or control oligo (miR-Neg). *:*p* < 0.05; **:*P* < 0.01; *t*-test; bars: SE, *n* = 6

To identify possible cancer-relevant targets of miR-4443, the TargetScan algorithm [9, 24] was used. Among the top-scored predicted targets of miR-4443, NCOA1 and TRAF4 have known roles in cell migration and cancer metastasis [35–39], and were chosen for validation. Exposure to leptin (at 100 ng/ml for 24 h), as well as overexpression of miR-4443 in HCT-116 cells (24 h post-transfection) resulted in significant (*p* < 0.05) down-regulation of endogenous NCOA1 and TRAF4 on the mRNA and protein levels (Fig. 3a–d). Insulin treatment (at 20 ng/ml) led to a similar down-regulation of NCOA1 and TRAF4 mRNA (Fig. 3a).

### NCOA1, TRAF4 are direct targets of miR-4443

To check if miR-4443 directly targets the 3′UTR region of NCOA1 and TRAF4 mRNAs, HCT-116 cells were co-transfected with miR-4443 mimic (or control oligos) and a secreted dual-reporter encoding vector, with or without the relevant 3′UTR (SecretePair from Genecopoeia). The Gaussia Luciferase signal was significantly suppressed by the co-transfected miR-4443 mimic, compared with the negative control oligo, in both NCOA1 and TRAF4 3′UTR-containing constructs. but not in a control plasmid lacking the 3′UTRs (Fig. 3g), supporting the predicted direct regulation of these mRNAs by miR-4443.

### The miR-4443 promoter/enhancer region contains the CCAAT motif, supporting regulation by C/EBPs

Since miR-4443 was up-regulated following both leptin and insulin treatment, we hypothesized that its promoter or enhancer regions may contain the CCAAT motif, which binds transcription factors from the CCAAT-enhancer-binding proteins (C/EBP) family, that act downstream of MEK1/2 in leptin and insulin signaling [3, 40–43] and were previously described as up-regulated by both insulin and leptin [44, 45]. To investigate this possibility, a 10 kb region of the human genomic sequence upstream of pre-miR-4443, which contains no other known genes on the same (plus) strand, was analyzed using the Cister algorithm [33]. Two high-score (0.99) matches for CCAAT motifs were found at positions 1903–18 and 1955–70 of the sequence, in addition to other adjacent cis-elements predicted to bind SP1 and E2F transcription factors that combine to form a predicted high probability enhancer region ~8 kb upstream of the miR-4443-encoding locus (Additional file 4: Figure S2). Additional binding motifs present on the opposite (minus) strand at the same locus, likely affect the expression of the adjacent CDC25A gene. This *in*-*silico* analysis suggests that miR-4443 is regulated by the MEK1/2 – C/EBP pathway downstream of both the insulin and leptin receptors, providing a possible explanation for the similar effects of exposure to insulin and leptin on miR-4443 and its downstream targets.

### The up-regulation of miR-4443 by leptin and insulin is attenuated by the inhibition of MEK1/2

**Fig. 4:**
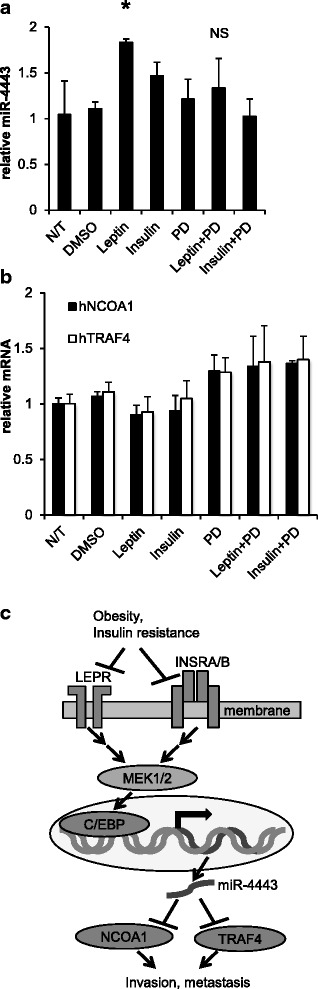
The effects of leptin and insulin on miR-4443 and NCOA1/TRAF4 are likely mediated by the MAPK pathway. **a**–**b** RT-qPCR for miR-4443 (**a**), or human NCOA1 and TRAF4 mRNAs (**b**), in HCT-116 cells, pre-incubated for 45 min, with or without a MEK inhibitor, PD-98059, an inhibitor of MEK activity (dissolved in DMSO) (10 μM), before being exposed to leptin (100 ng/ml) or insulin (20 ng/ml) for 24 h. Controls included the appropriate concentration of DMSO (less than 0.1 %), PD alone and leptin or insulin alone. *p < 0.05, significantly different from N/T, *t*-test; NS – not significantly different from N/T; bars: SD from triplicates. **c** Scheme, proposed mechanism of miR-4443-mediated signaling downstream of leptin and insulin

To assess if MEK1/2 indeed regulate miR-4443 downstream of leptin and insulin signaling, HCT-116 cells were pre-incubated for 45 min with or without a MEK inhibitor, PD-98059 (10 μM), before being exposed to leptin (100 ng/ml) or insulin (20 ng/ml) for 24 h. The inhibitor attenuated the leptin-induced up-regulation of miR-4443 (Fig. 4a). A similar trend was observed with insulin, although the up-regulation was modest (Fig. 4a). Co-treatment with PD-98059 also abolished the down-regulation of NCOA1 and TRAF4 mRNA levels by leptin in these cells (Fig. 4b). These results support the notion that miR-4443 is regulated by the MEK1/2 – C/EBP pathway downstream of both the insulin and leptin receptors, although it is impossible to discount other, miR-4443-independent signaling pathways that could affect the levels of NCOA1 and TRAF4 downstream of these receptors.

## Discussion

Using three CRC-derived lines as a cellular model, we have identified a signaling pathway that integrates insulin and leptin signaling in the activation of MEK1/2 and leads to up-regulation of miR-4443, which in turn down-regulates NCOA1 and TRAF4, possibly causing tumor suppression and decreased cell invasion. Acquired resistance to leptin and/or insulin is likely to interfere with this signaling and increase the risk of metastatic cancer (scheme, Fig. 4c); the lack of reaction of DLD-1 cells to leptin in our study illustrates such dysfunction on the cellular level. Our results suggest that acquired resistance to leptin and insulin, rather than high levels of these hormones in the circulation, may have a causal role underlying the epidemiological correlation between obesity and cancer risk.

Several studies have shown that the effects of leptin on the behavior of CRC-derived cells are complex and vary, based on the specific origin of the cell lines and their particular phenotypic and molecular repertoire [46–49]. For instance, when exposed to leptin, *enhanced* motility and invasion were observed in LS174T and HM7 cell lines [47] and *increased* proliferation was reported in several lines, including HCT-116 [46, 48], while in another study, no change in proliferation was observed following leptin treatment [49]. Although differences in protocols are a common cause for divergence between studies, this inconsistency could also be caused by genetic differences. The genetic variability between – and within – cell lines may be caused not only by the genetic background of their respective origin, but also stem from the independent selection that cell line batches undergo in culture (see, for example, [50]). Anticipation of such differences is the main reason for choosing to perform the study in three different cell lines (HCT-116, HT-29 and DLD-1). Indeed, our results show that one of the lines (DLD-1) exhibited abnormal expression of LEPR, and in agreement with our proposed regulatory pathway, miR-4443 was not significantly affected by leptin treatment in this line. These differences further underscore the importance of thorough molecular characterization in the context of research as well as in diagnostics. The miRNA expression profile is an increasingly recognized aspect of such molecular characterization.

In our study, miR-4443 greatly contributed to the observed correlation between the effects of insulin and leptin exposure on miRNA expression profiles of CRC-derived cell lines. However, other miRNAs are likely to be regulated by the MAPK cascade downstream of both insulin and leptin, which would explain the broad correlation between their effects.

## Conclusion

Our results suggest that miR-4443 mediates a novel mechanism by which the metabolic state of the organism may influence the risk of tumorigenesis. Further studies are required to ascertain the significance of this signaling pathway in human patients and in animal models of obesity and cancer.

## Additional files

Additional file 1: Table S1. List of primers used. (XLSX 10 kb)
Additional file 2: Figure S1. Hierarchically clustered heat map for leptin-induced changes in miRNA expression, in HCT-116, HT-29 and DLD-1 cells. (TIFF 5506 kb)
Additional file 3: Table S2. Nanostring array results - all miRNA expression profiling data. (XLSX 174 kb)
Additional file 4: Figure S2. Cister algorithm prediction of a cis-element cluster in *hsa*-*mir*-*4443* promoter sequence. (PPTX 88 kb)

## Abbreviations

CRC: Colorectal cancer
LEPR: Leptin receptor
miRNA: microRNA
RT-qPCR: Reverse transcription and quantitative polymerase chain reaction
