# Supplementary figures and images for: Leptin and insulin up-regulate miR-4443 to suppress NCOA1 and TRAF4, and decrease the invasiveness of human colon cancer cells
Source: BMC Cancer. 2016 Nov 14;16:882. doi: 10.1186/s12885-016-2938-1 (PMC5109693; doi:10.1186/s12885-016-2938-1)

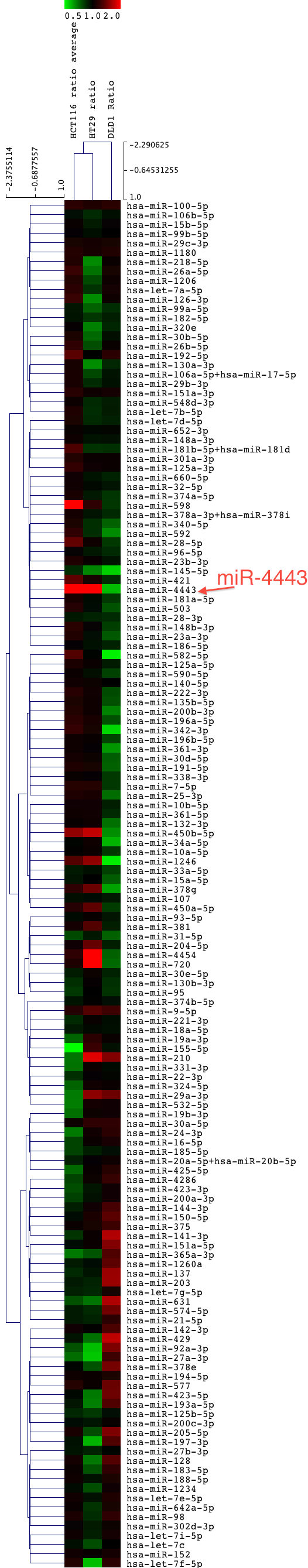

Supplement: Additional file 2: Figure S1. — Hierarchically clustered heat map for leptin-induced changes in miRNA expression, in HCT-116, HT-29 and DLD-1 cells. (TIFF 5506 kb) [file 12885_2016_2938_MOESM2_ESM.tiff]
