# Supplementary material for: Leptin and insulin up-regulate miR-4443 to suppress NCOA1 and TRAF4, and decrease the invasiveness of human colon cancer cells
Source: BMC Cancer. 2016 Nov 14;16:882. doi: 10.1186/s12885-016-2938-1 (PMC5109693; doi:10.1186/s12885-016-2938-1)
Supplement: Additional file 4: Figure S2. — Cister algorithm prediction of a cis-element cluster in hsa-mir-4443 promoter sequence. (PPTX 88 kb) [file 12885_2016_2938_MOESM4_ESM.pptx]

## Slide 1
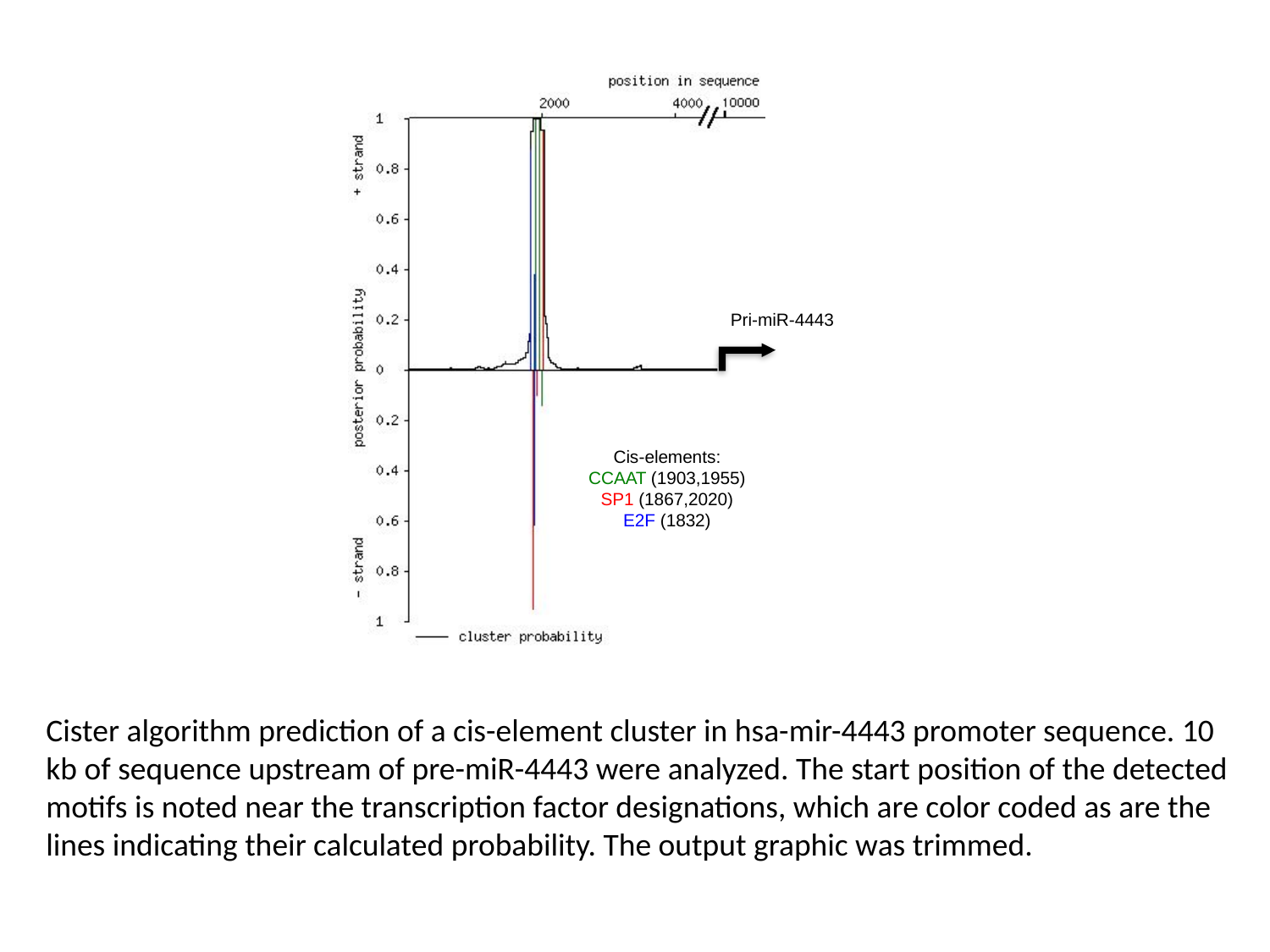

Pri-miR-4443
Cis-elements:
CCAAT (1903,1955)
SP1 (1867,2020)
E2F (1832)
Cister algorithm prediction of a cis-element cluster in hsa-mir-4443 promoter sequence. 10 kb of sequence upstream of pre-miR-4443 were analyzed. The start position of the detected motifs is noted near the transcription factor designations, which are color coded as are the lines indicating their calculated probability. The output graphic was trimmed.
